# Best Evidence Summary for the Improvement and Management of Disorders of Consciousness in Patients With Severe Brain Injury

**DOI:** 10.1002/brb3.70260

**Published:** 2025-01-09

**Authors:** Miaoyuan Lin, Qiongna Lu, Sheng Yu, Wenjuan Lin

**Affiliations:** ^1^ Department of Neurosurgery Shenzhen Nanshan People's Hospital Shenzhen Guangdong People's Republic of China; ^2^ School of Health Guangzhou Vocational and Technical University of Science and Technology Guangzhou Guangdong People's Republic of China

**Keywords:** awareness, brain injury, disorders of consciousness, evidence summary

## Abstract

**Background and Purpose:**

The treatment effect of consciousness after brain injury is currently uncertain. Thus, this study aimed to retrieve the evidence from neurologists around the world on the management of consciousness disorders in patients with severe brain injury and evaluate and summarize the evidence, providing the guidance on the related management for clinicians.

**Methods:**

Following the evidence summary report standard of Fudan University Center for Evidence‐Based Nursing, clinical guidelines, expert consensuses, systematic reviews, and evidence summaries were systematically retrieved from UpToDate; BMJ Best Practice; Guidelines International Network; the Cochrane Library; Embase; PubMed; Sinomed; Web of Science; CNKI; WanFang database; American Academy of Neurology (AAN); American Congress of Rehabilitation Medicine (ACRM); European Academy of Neurology; and National Institute on Disability, Independent Living, and Rehabilitation Research (NIDILRR). The publishing timeline for articles was limited from January 2017 to January 2024.

**Results:**

Fourteen articles were finally identified. The 26 best pieces of evidence were recommended by inducting and integrating the evidence from these articles, covering the following seven aspects: consciousness assessment, multidisciplinary team, intervention in facilitating arousal, sensory stimulation programs, drug administration, rehabilitation program, and prevention of complications.

**Conclusion:**

This study summarized the evidence of consciousness management in patients with brain injury, providing guidance for clinicians to develop and apply those interventions to improve the patient's clinical outcomes and quality of life. In addition, relevant factors such as the clinical environment and cooperation with the patient's family members should be evaluated and adjusted before applying such evidence. Future studies should focus on more targeted randomized clinical trials.

## Graphical abstract

This study summarized the evidence of consciousness management in patients with brain injury through evidence summary report standard, which provides the 26 best evidences, covering the following seven aspects: consciousness assessment, multidisciplinary team, intervention in facilitating arousal, sensory stimulation programs, drug administration, rehabilitation program, and prevention of complication.

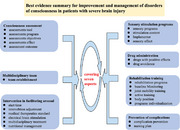

## 1 Introduction

Disorders of consciousness (DoCs) are the result of multidimensional dysfunction of the complex cerebral pathways responsible for awareness and wakefulness, with brain injury (BI) representing the most common cause (Farisco et al. 2024; Squitti et al. 2023). Survivors of DoCs need continuous professional medical care and have to transition between multiple postacute care facilities, posing a heavy financial burden on families and society and affecting the quality of their life (Zheng et al. 2023; Muehlschlegel et al. 2024). BI damages the patient's brainstem reticular formation, affecting cortical function transmission and integration and changing cognitive and physical function, as well as the central nervous system. Many such patients endure long‐term disabilities (physically, cognitively, or both), particularly those with moderate‐to‐severe traumatic BI (TBI; msTBI; Farisco et al. 2024). In the United States, msTBI contributes to approximately 30% of all injury‐related deaths, that is, annual mortality of 61,000 Americans (Muehlschlegel et al. 2024). Although the overall morbidity of patients with BI is only 13/100,000, there is an alarming mortality rate of up to 50%, whereas the remaining 10%–15% of patients develop prolonged DoC (pDoC) among high‐risk patients (Capizzi, Woo, and Verduzco‐Gutierrez 2020).

pDoC is defined as a coma state lasting > 28 days. When a patient does not recover their consciousness in a given time, they will transition into the minimally conscious state (MCS) or unresponsive wakefulness syndrome (UWS) based on the degree of the impairment of systems related to wakefulness and awareness, with an average survival of only 2–5 years (Zheng et al. 2023; Giacino et al. 2020; Zhao et al. 2023; Ma et al. 2023). Regarding structural and functional neuroimaging, patients in MCS and vegetative state (VS) showed differences in corticocortical and corticothalamic connectivity (Giacino et al. 2020). In MCS patients, neural pathway plasticity and functional reorganization demonstrated a better capacity than in UWS patients, underscoring the need for early therapeutic to improve consciousness (Giacino et al. 2020; Lu et al. 2021).

Patients emerging from unconsciousness showed signs of awareness in the earlier stages, emphasizing the evolution of awareness recovery (Royal College of Physicians 2022). Therefore, more effective and appropriate approaches are required for DoCs consciousness rehabilitation. Many treatments, including pharmacotherapy, hyperbaric oxygen therapy, and neuromodulation techniques, are currently used to manage DoCs following BI, although with greatly different efficacy (Lu et al. 2021; Briand et al. 2020; Lai et al. 2020; Bourdillon et al. 2019). Achieving consciousness improvement in patients with DoCs is an issue necessitating exploration for standardization, systematization, and facilitation of injury recovery during the rehabilitation period (Farisco et al. 2024). Therefore, this study comprehensively searched for studies on the management of consciousness improvement in patients with DoCs, systematizing and providing the best scientific evidence for clinical practice.

## 2 Methods

Using the reporting standard of evidence summary report of the Fudan University Center for Evidence‐Based Nursing guidance, this evidence summary established the research problem, retrieved relevant literature, screened and evaluated the literature, summarized and graded evidence, and presented practical suggestions (Peng et al. 2024).

### 2.1 Establishing the Research Problem

This study used the PIPOST model to solve the first step in the reporting standard of evidence summary—defining the research problem—as follows: (1) population (P): patients with critical BI and resulting of DoCs aged ≥ 18 years; (2) intervention (I): appropriate DoCs management in patients with BI, including assessment, treatment care, pharmacotherapy, rehabilitation training, and nursing; (3) professional (the second P): medical personnel; (4) outcome (O): improvement in consciousness levels and reduction in the complication incidence; (5) setting (S): the neurological unit, intensive care unit, and rehabilitation centers; and (6) type of evidence (T): evidence from clinical guidelines, expert consensus, evidence summary, best practice, and high‐quality system reviews.

### 2.2 Searching Databases and Search Strategy

According to the top‐down “6S” pyramid evidence model, the following databases were used to retrieve relevant articles: UpToDate, BMJ, JBI, Guidelines International Network, Embase, The Cochrane Library, Registered Nurses Association of Ontario, PubMed, Sinomed, Web of Science, CBM, CNKI, WanFang, VIP, Chinese Medical Journal Full‐Text Database, European Society for Clinical Nutrition and Metabolism website, and the American Society for Parenteral and Enteral Nutrition website. Furthermore, the related references were tracked. The search time frame was set from January 2017 to January 2024.

The search strategy was created using a combination of subject and free words. The terms for the search were (brain injuries OR traumatic brain injury OR brain trauma OR acquired brain injury OR head injury OR head trauma OR craniocerebral trauma OR traumatic OR TBI) AND (disorders of consciousness OR unconsciousness OR neuropsychiatric disorder OR consciousness dysfunction OR consciousness deficit OR coma OR vegetative state OR minimally conscious state) AND (clinical decision making OR guidelines OR evidence summaries OR best practices OR recommended practices OR expert consensus OR systematic reviews).

### 2.3 Study Inclusion and Exclusion Criteria

The inclusion criteria were as follows: (1) patients with critically BI aged ≥ 18 years as study subjects; (2) articles focused on improving DoCs; (3) clinical guidelines, expert consensus, best decision‐making, expert recommendation evidence summary, and systematic evaluation articles; and (4) the latest guideline versions (updated or revised).

The exclusion criteria were as follows: (1) abstract‐only articles or articles with unavailable full‐text; (2) low‐quality articles; (3) articles with published duplicates or nonoriginal guidelines; and (4) articles written in language other than English and Chinese.

### 2.4 Literature Screening

Duplicate literature was excluded through the EndNote software. Following the inclusion and exclusion criteria, two researchers independently retrieved the obtained literature. Primary screening was conducted by reading the title, abstract, and keywords. Then, the full texts were read for the secondary screening, followed by evaluating the quality of the selected articles. For the controversial literature, a third specialist with evidence‐based research training experience was responsible for decisions until a consensus was reached. Finally, the study characteristics were extracted in a double‐blind manner.

### 2.5 Quality Evaluation of the Literature

The appropriate tools were applied to evaluate different types of article quality. The Appraisal of Guidelines for Research and Evaluation II (AGREE II), which was made by scholars from 13 different countries and updated in 2017, was used to assess guidelines. Systematic reviews were evaluated by the systematic evaluation tool of the Assessment of Multiple Systematic Reviews 2 (AMSTAR 2). The Expert Consensus Evaluation Criteria of JBI Evidence‐Based Health Care Centers was adopted to evaluate expert consensus in this study. In addition, no specialized assessment instrument was used for the quality evaluation of evidence summaries and clinical decisions. Consequently, these articles were judged by tracing the original document of each evidence source and selecting the corresponding tool (Gao et al. 2024).

### 2.6 Evidence Extraction and Summary

Evidence from the literature was extracted and summarized based on practicability, appropriateness, meaningfulness, and usefulness evaluation principles. Integration was conducted for conflicting conclusions. The following inclusion principles were prioritized: high‐quality evidence, evidence‐based evidence, and the most recent published and authoritative articles.

### 2.7 Evidence Level and Recommendation Level Criteria

The evidence grading level was evaluated using the 2014 JBI Evidence Pre‐Classification and Oxford Centre for Evidence‐Based Medicine (OCEBM) Evidence Recommendation Level System (The Joanna Briggs Institute 2014; Romanowski et al. 2020). In the 2014 JBI Evidence Pre‐Classification, the evidence level is divided into five levels according to the evidence design from the highest rank to the lowest rank as follows: Levels 1, 2, 3, 4, and 5. The more rigorous the research design, the higher the evidence level. Level 1 represents randomized controlled trials (RCTs) and meta‐analyses of RCTs, while Level 2 is a quasiexperimental study. Level 3 is an observational‐analytical research, Level 4 is an observational‐descriptive study, and Level 5 includes expert opinions and basic research (Romanowski et al. 2020). Subsequently, the evidence was recommended by using the OCEBM Evidence Recommendation criteria. The higher the feasibility, effectiveness, clinical significance, and appropriateness of the evidence, the higher the evidence recommendation level (Zhang et al. 2024). Grade A (strong evidence) and Grade D (conflicting evidence) are at the top and bottom in the rank order, respectively. Grade A is consistent with level studies, Grade B is consistent with Level 2 or 3 studies or extrapolations from Level 1 studies. Grade C includes Level 4 studies or extrapolations from Level 2 or 3 studies, whereas Grade D is Level 5 evidence or troublingly inconsistent or inconclusive studies of any level (Zhang et al. 2024).

## 3 Results

### 3.1 Search Results

**FIGURE 1:**
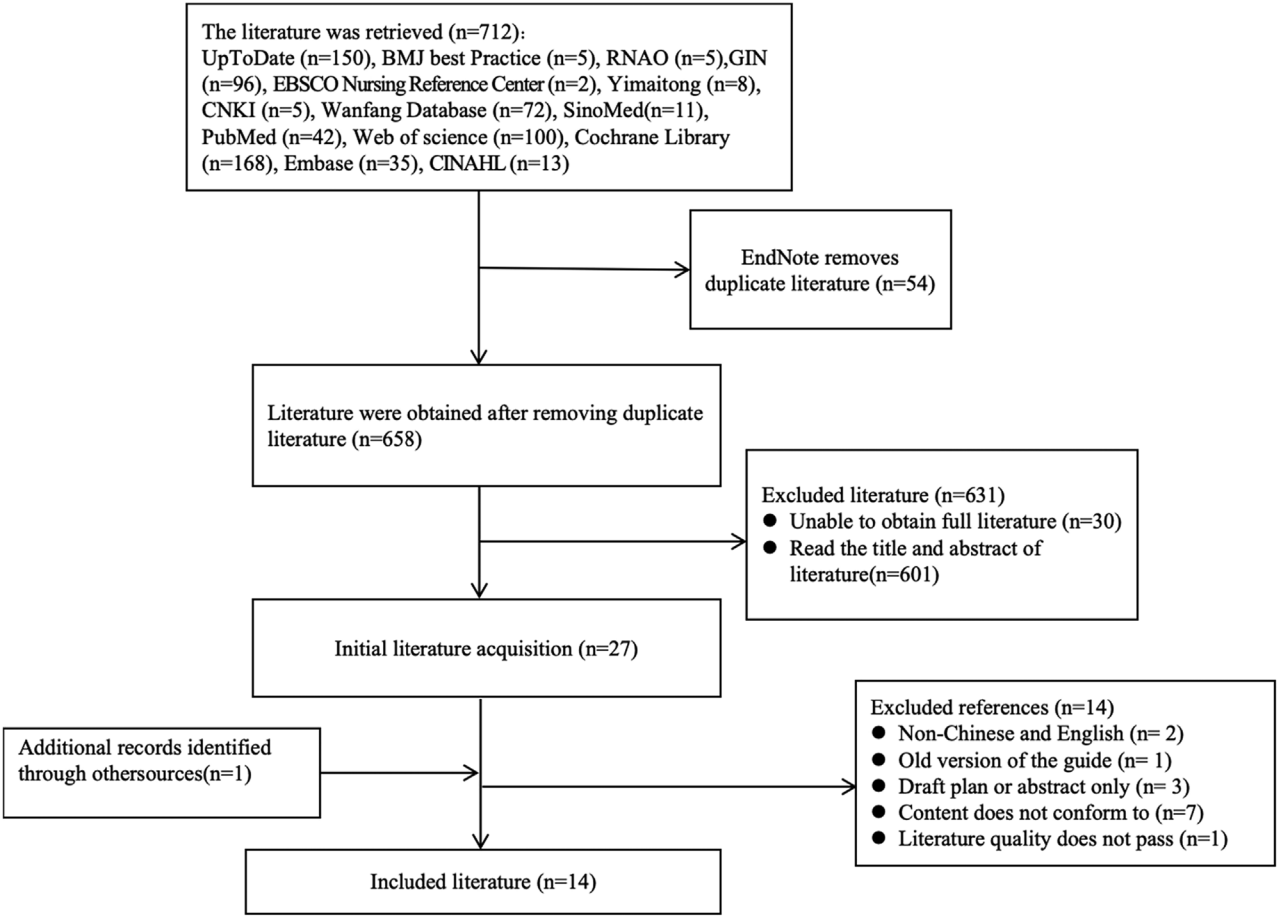
Flow diagram of literature screening. The basic characteristics of the included literature are shown in Table 1.

Figure 1 demonstrates the PRISMA flow diagram–based literature screening. A total of 712 articles were obtained, with 658 articles left after duplicate removal through the Endnote software. For these articles, titles and abstracts were reviewed, with 27 studies chosen for full‐text screening. Finally, 14 articles were included in the final analysis after excluding literature without full texts and unrelated items that did not meet the inclusion criteria. Since two guidance (Giacino et al. 2018; Giacino, Katz, and Schiff 2019) tools were from the same association and authors, we chose the latest updated guidance (Giacino, Katz, and Schiff 2019). These 14 articles comprised six guidelines, five expert consensus, and three systematic reviews.

### 3.2 Quality Evaluation of the Included Literature

#### 3.2.1 Quality Evaluation for Guidelines

Six guidelines were included in the summary, with evaluation results shown in Table 2. As demonstrated in Table 2, all guidelines had intraclass correlation of coefficient (ICC) values > 0.75, indicating the high reliability and good congruence of the literature.

**TABLE 1:** General characteristics of the included literatures (*n* = 14).

Included literature	Year of publication (year)	Literature source	Type of literature	The literature theme
Zheng et al. (2023)	2023	PubMed	Expert consensus	Clinical identification and management of disorders of consciousness after acquired brain injury
Giacino, Katz, and Schiff (2019)	2019	Web of Science	Guideline	Practice parameter on persistent vegetative state and the definition of MCS and provide care recommendations for patients with pDoC
Kondziella et al. (2020)	2022	PubMed	Guideline	Coma and disorders of consciousness diagnosis
China Association of Rehabilitation of Disabled Persons, China Association of Rehabilitation Medicine, and China Rehabilitation Research Center (2023)	2023	Wanfang database	Expert consensus	Prolonged disorders of consciousness rehabilitation
Ni et al. (2018)	2018	CNKI	Expert consensus	Expert consensus on the management of neurosurgical critical illness rehabilitation in China
Giacino et al. (2020)	2020	PubMed	Guideline	Clinical practice guidelines for rehabilitation care standards in brain injury rehabilitation
Group of Disorders of Consciousness, and Conscious‐Promotion, Professional Committee of Neurorepair of Chinese Medical Doctor Association (2020)	2020	Chinese Medical Association	Expert consensus	Chinese expert consensus on diagnoses and treatments of prolonged disorders of consciousness
Arai et al. (2021)	2021	PubMed	Systematic review	Conscious effects of transcranial magnetic stimulation and electroencephalography
Burgos et al. (2018)	2018	PubMed	Guideline	Guideline clinical nutrition in neurology
Li et al. (2020)	2020	Web of Science	Systematic review	Sensory stimulation
Zuo et al. (2021)	2021	PubMed	Systematic review	Family‐centered sensory and affective stimulation interventions
Togher et al. (2023)	2023	PubMed	Guideline	Traumatic brain injury cognitive rehabilitation
Ponsford et al. (2023)	2023	Web of Science	Guideline	Cognitive attention disorder rehabilitation
Ashford et al. (2021)	2021	Update	Expert consensus	Clinical practice consensus for managing physical well‐being on pDoC

**TABLE 2:** Methodological quality evaluation results of the clinical guidelines (*n* = 6).

Guideline	Standardized scores in various domains (%)									
	Scope and purpose	Stakeholder involvement	Rigor of development	Clarity of presentation	Applicability	Editorial independence	≥ 60% field number (*n*)	≥ 30% field number (*n*)	ICC	Recommendation level
Giacino, Katz, and Schiff (2019)	0.938	0.896	0.667	0.958	0.417	0.958	5	6	0.914	B
Kondziella et al. (2020)	0.958	0.596	0.746	0.950	0.762	0.967	5	6	0.883	B
Giacino et al. (2020)	0.967	0.950	0.883	0.913	0.804	0.983	6	6	0.810	A
Burgos et al. (2018)	0.958	0.971	0.979	0.971	0.742	0.988	6	6	0.904	A
Togher et al. (2023)	0.896	0.704	0.804	0.967	0.888	0.896	6	6	0.960	A
Ponsford et al. (2023)	0.908	0.871	0.754	0.946	0.913	0.983	6	6	0.868	A

#### 3.2.2 Quality Evaluation for Expert Consensus

The five included expert consensuses had high quality with rational research designs (Table 3). For the expert consensuses by Zheng et al. (2023), Group of Disorders of Consciousness and Conscious‐Promotion, Professional Committee of Neurorepair of Chinese Medical Doctor Association (2020), and Ashford et al. (2021), each item was answered as “Yes.” Although the fifth item was evaluated as “unclear” in the expert consensus by the China Association of Rehabilitation of Disabled Persons, China Association of Rehabilitation Medicine, China Rehabilitation Research Center (2023) and Ni et al. (2018), the remaining items were assessed as “Yes.” The related quality evaluation was high, and the overall quality assessment was still high.

**TABLE 3:** Quality evaluation results of expert consensus (*n* = 5).

Items	Zheng et al. (2023)	China Association of Rehabilitation of Disabled Persons, China Association of Rehabilitation Medicine, and China Rehabilitation Research Center (2023)	Ni et al. (2018)	Group of Disorders of Consciousness, and Conscious‐Promotion, Professional Committee of Neurorepair of Chinese Medical Doctor Association (2020)	Ashford et al. (2021)	
1. Is the source of the comment cleared?	Yes	Yes	Yes	Yes	Yes	
**2**. Is the source of comment from the expertise field?	Yes	Yes	Yes	Yes	Yes	
3. Does comment involve conflict of interest?	Yes	Yes	Yes	Yes	Yes	
4. Has the comment been verified, and is there logic in the stated expression?	Yes	Yes	Yes	Yes	Yes	
5. Are there reference sources?	Yes	Yes	Yes	Yes	Yes
6. Is there logical defense for the incongruence with extant literature?	Yes	Unclear	Unclear	Yes	Yes

#### 3.2.3 Quality Evaluation for Systematic Reviews

Table 4 shows the quality evaluation results of three included systematic reviews. Accordingly, no items or only two noncritical items were inconsistent in all systematic reviews, indicating the high quality of the evaluated systematic reviews.

**TABLE 4:** Quality evaluation results of systematic review (*n* = 3).

Items	Arai et al. (2021)	Li et al. (2020)	Zuo et al. (2021)
1. Identifiable study questions	Yes	Yes	Yes
2. Logical inclusion and exclusion criteria	Unclear	Yes	Yes
3. Appropriate retrieval strategy	Yes	Yes	Yes
4. Comprehensive databases	Yes	Yes	Yes
5. Appropriate quality evaluation method	Yes	Unclear	Yes
6. Independent quality assessment completed by two or more researches	Yes	Yes	Yes
7. Reasonable production of point	Yes	Yes	Yes
8. Appropriate methodology for different types of studies	Yes	Yes	Yes
9. Report of bias evaluation	Yes	Unclear	Yes
10. Option based on results	Yes	Yes	Yes
11. Appropriate directions for future research	Yes	Yes	Yes

### 3.3 Summary and Description of Evidence

Using literature summary and evaluation, 26 pieces of best available evidence from seven areas were described. These included the assessment of consciousness levels, multidisciplinary teams, interventions in facilitating arousal, sensory stimulation programs, drug administration, rehabilitation programs, and the prevention and management of complications (Table 5).

**TABLE 5:** Summary of evidences for improvement and management of the state of consciousness brain injury patients.

Category	Content of evidence	Evidence level	Recommendation level
Consciousness assessment	1. Assessment tool: The Coma Recovery Scale–Revised (CRS‐R) is recommended, which is used five times a week, preferably daily, with repeated evaluation to select the optimal assessment result (Zheng et al. 2023; Giacino, Katz, and Schiff 2019; Kondziella et al. 2020; China Association of Rehabilitation of Disabled Persons, China Association of Rehabilitation Medicine, China Rehabilitation Research Center 2023; Ni et al. 2018)_._	Level 2	A
	2. Assessment program: Relevant factors that are contributing to body condition and limitations should be considered whenever possible, including personal factors (age), injury‐related factors (traumatic or nontraumatic), and other factors that may affect the rehabilitation and treatment efficacy (duration of DoCs; Giacino, Katz, and Schiff 2019; China Association of Rehabilitation of Disabled Persons, China Association of Rehabilitation Medicine, China Rehabilitation Research Center 2023).	Level 5	A
	3. Assessment objectives: The assessment should be multidimensional, using a multimodal or multidisciplinary framework that integrates standardized clinical evaluation, advanced neuroimaging, and electrophysiological technologies to reduce clinical misdiagnosis and improve prognostic predictions (Zheng et al. 2023; China Association of Rehabilitation of Disabled Persons, China Association of Rehabilitation Medicine, China Rehabilitation Research Center 2023; Ni et al. 2018; Giacino et al. 2020).	Level 5	A
	4. Assessment effect: Assessments should be repeated regularly, as performance reaches the ceiling on a particular instrument, transitioning to measures capable of capturing more complex functions (Giacino et al. 2020).	Level 5	A
	5. Assessment outcome: The true state of consciousness depends on the best revealed by assessment approaches (Kondziella et al. 2020).	Level 5	A
Multidisciplinary team	6. Team establishment: A multidisciplinary rehabilitation team is formed, with the neurologist or rehabilitation physician as the team leader and other specialized members included based on the patient's physical needs, the course of the disease, or the actual situation of the rehabilitation organization (China Association of Rehabilitation of Disabled Persons, China Association of Rehabilitation Medicine, China Rehabilitation Research Center 2023; Giacino et al. 2020).	Level 5	A
Interventions in facilitating arousal	7. Starting time: Interventions should be started as early as 24 hours after neurological stabilization while identifying patient and family intervention preferences, providing the most significant treatment effect in 3 months (Giacino, Katz, and Schiff 2019; Kondziella et al. 2020; China Association of Rehabilitation of Disabled Persons, China Association of Rehabilitation Medicine, China Rehabilitation Research Center 2023; Ni et al. 2018).	Level 3	B
	8. Intervention adjustment: Environmental factors (e.g., positioning, lighting, time of day, level of stimulation, distractions, and restraint) should regularly assess the potential impact of arousal and neurocognitive performance while making adjustments accordingly to optimize interpersonal interactions and participation (Giacino et al. 2020).	Level 5	A
	9. Medical therapeutics standard: Treatments addressing patients' etiologies and complications to promote the restoration and reconstruction of neural networks of consciousness is recommend, for example, cranial reconstructions should be performed as early as possible within 3–6 months after injury (China Association of Rehabilitation of Disabled Persons, China Association of Rehabilitation Medicine, China Rehabilitation Research Center 2023; Ni et al., 2018).	Level 5	A
	10. Electrical brain stimulation: It is mainly categorized into noninvasive and invasive brain stimulation. It could be performed in patients with stable disease or subsided cerebral edema, after the conventional rehabilitation is deemed ineffective (Group of Disorders of Consciousness and Conscious‐Promotion, Professional Committee of Neurorepair of Chinese Medical Doctor Association 2020; Arai et al. 2021).	Level 3	C
	11. Multidisciplinary treatment: Based on the current evidence, providing comprehensive multidisciplinary treatment including physical factor therapy, hyperbaric oxygen therapy, herbal therapy, and swallowing therapy to prevent the progression of DoCs is recommended (China Association of Rehabilitation of Disabled Persons, China Association of Rehabilitation Medicine, China Rehabilitation Research Center 2023; Group of Disorders of Consciousness and Conscious‐Promotion, Professional Committee of Neurorepair of Chinese Medical Doctor Association 2020).	Level 5	B
	12. Nutritional management: Patients should receive nutritional goal management, including the assessment of the baseline nutritional state, symptoms of feeding intolerance, nutritional pathways, food types, and BMI target. The energy target should be 25–30 kcal/(kg/d) and the protein target should be 1.2–2.0 g/(kg/d). If fever and increased muscle tone occur, the energy target should be increased following a reasonable supply (China Association of Rehabilitation of Disabled Persons, China Association of Rehabilitation Medicine, China Rehabilitation Research Center 2023; Burgos et al. 2018).	Level 5	A
Sensory stimulation	13. Sensory programs: Patients should receive sensory stimulation programs, including sight, hearing, smell, taste, touch, and vestibular senses training, several times a day for 10–20 min each time (Li et al. 2020).	Level 5	A
	14. Stimulus content: Playing familiar and favorite songs, films, and TV episodes and instructing family members to make occasional contact with the patient's limbs to soothe them are recommended, with stimulation combined with emotional or autobiographical content being more effective (Li et al. 2020; Togher et al. 2023).	Level 5	B
	15. Implementers: The improvement of consciousness with the stimulation performed by family members is greater than that with the stimulation implemented by nurses, resulting in better awakening time, awakening rate, and satisfaction rate (Zuo et al. 2021).	Level 2	A
	16. Sensory effect: The combination of auditory and tactile stimulation or multisensory stimulation is the most effective and easiest to implement for improving the level of consciousness (China Association of Rehabilitation of Disabled Persons, China Association of Rehabilitation Medicine, China Rehabilitation Research Center 2023; Zuo et al. 2021).	Level 1	B
Drug administration	17. Drugs with positive effects: Drugs acting on dopaminergic, catecholaminergic, and cholinergic systems (amantadine, bromocriptine, and naloxone) should be prescribed as these medications may fasten functional recovery and reduce disability early during recovery (Giacino, Katz, and Schiff 2019; Ni et al. 2018; Giacino et al. 2020; Group of Disorders of Consciousness and Conscious‐Promotion, Professional Committee of Neurorepair of Chinese Medical Doctor Association 2020).	Level 2	B
	18. Drug avoidance: The use of neuroleptics and benzodiazepines to treat agitation or aggression should be minimized as these medications may slow recovery after brain injury and may affect consciousness. When medications are needed, monitoring the impact on agitation and consciousness using standardized tools is recommended (Ponsford et al. 2023).	Level 5	A
Rehabilitation training	19. Rehabilitation programs: Rehabilitation training involving the family should be provided to identify sensory stimulation. The patient's ability to perceive similarities and differences, limits of scope, interpersonal distancing, and judgment and expansion of speech and behavior is trained and reinforced simultaneously depending on the injury (Giacino et al. 2020).	Level 5	A
	20. Baseline Monitoring: Monitoring recovery in individual patients is recommended. Validated measures should be used to establish the level of performance at baseline, rate and trajectory of recovery, degree of disability, and response to individualized treatment (Giacino et al. 2020).	Level 5	A
	21. Joint mobility training: Passive training is recommended twice a day, with each session lasting 20 min, for maintaining the range of motion of the joints. Patients with muscle spasms should receive antispasmodic drugs and wear rehabilitation aids (China Association of Rehabilitation of Disabled Persons, China Association of Rehabilitation Medicine, China Rehabilitation Research Center 2023; Group of Disorders of Consciousness and Conscious‐Promotion, Professional Committee of Neurorepair of Chinese Medical Doctor Association 2020).	Level 2	B
	22. Active training: Patients with partial recovery of consciousness should be assisted in active training (assisted seated training or stationary standing training at different angles on an upright bed, gradually increasing the angle). Furthermore, the period of weekly adaptive training twice a day, with each session lasting 20 min, is recommended (Group of Disorders of Consciousness and Conscious‐Promotion, Professional Committee of Neurorepair of Chinese Medical Doctor Association 2020).	Level 3	B
	23. Body position: Patients should sleep on a pressure‐relieving mattress for a long period, change position regularly, and maintain a functional position (China Association of Rehabilitation of Disabled Persons, China Association of Rehabilitation Medicine, China Rehabilitation Research Center 2023; Ashford et al. 2021).	Level 5	A
	24. Programs individualization: Clinicians should make interventions according to individual needs, accounting for the level of consciousness severity and stage of recovery during treatment decisions (Group of Disorders of Consciousness and Conscious‐Promotion, Professional Committee of Neurorepair of Chinese Medical Doctor Association 2020).	Level 5	A
Prevention of complications	25. Complication prevention: The specialist assessment of medical complications by a skilled multidisciplinary team should be performed, comprising prevention, management, and the monitoring of the general state of health (including skin integrity, bed positioning, and abnormal posturing). If necessary, interventions should be adjusted to account for the results of the multidisciplinary team's evaluation (Giacino et al. 2018; Giacino et al. 2020; Ashford et al. 2021).	Level 5	A
	26. Nursing plan: The updated weekly and streamlined nursing care plan shall be considered for all patients within admission, possibly to reduce the burden of future care. The programs include adequate nutrition, respiratory hygiene and aspiration risk, bladder and bowel management, skin integrity, contracture prevention, positioning and tone management, prevention of venous thrombosis, and optimizing sleep/wake patterns (Giacino et al. 2020).	Level 5	A

## 4 Discussion

DoCs affect the prognosis and outcome of patients with BI and reduce the quality of life of their families. Timely and effective medical treatment programs are the key to improving consciousness level and rehabilitation prognosis for DoCs patients (Group of Disorders of Consciousness and Conscious‐Promotion, Professional Committee of Neurorepair of Chinese Medical Doctor Association 2020). Nonetheless, a harmonized clinical decision‐making methodology is widely used to improve the consciousness level in DoCs patients (Zheng et al. 2023). The management and improvement of awareness have always been the problem in patients with critical BI. Therefore, this study summarized 26 pieces of the best available evidence for the rehabilitation management of patients with critical BI and DoCs through an evidence‐based literature search and rigorous evidence extraction, providing scientifically standardized and operable practice protocols.

### 4.1 Consciousness Assessment

The obtainment of postinjury clinical findings during the first 7 days decided the decision‐making specific to the intervention (Giacino et al. 2020). For the first aspect, the consciousness level of a DoCs patient should be evaluated as soon as possible (Farisco et al. 2024). As demonstrated in Table 5, the first to the fifth of evidence summarized the evaluation time, tools, and method for consciousness state assessment in DoCs patients. The key goals of DoCs management in the acute phase are to prevent misinterpretation, recognize reversible harm, and provide an accurate prognosis of awakening (Zheng et al. 2023). Obtaining timely and accurate assessment results represented the rationale for individualized intervention decisions.

Such an approach has some advantages to using a repeated Coma Recovery Scale–Revised (CRS‐R) examination technique, with a minimum of six repetitions in patients with TBI and six in patients without TBI, which can reduce misdiagnosis within 1 week (Kondziella et al. 2020; China Association of Rehabilitation of Disabled Persons, China Association of Rehabilitation Medicine, China Rehabilitation Research Center 2023; Ni et al. 2018). Consciousness level evaluation repeated regularly can not only predict patient prognosis but also be beneficial to embrace prime time for the awakening of patients with BI to reduce the occurrence of DoCs (Wang et al. 2020). This point was also mentioned in previous studies.

Currently, unified standards for the accurate assessment of consciousness levels, which are predominantly based on brain function symptoms such as auditory, vision, motor, language, and eye activity, are lacking. Among multiple screening tools for clinical consciousness assessment, the CRS‐R score is the most widely used. The assessment of consciousness level should be multidimensional, multimodal, and multidisciplinary, integrating clinical assessment, advanced neuroimaging, and electrophysiology techniques. However, the level of evidence from expert consensus is low.

The European and the US guidelines recommend basing clinical diagnosis through the CRS‐R tool. Meanwhile, three validated tools such as the Wessex Head Injury Matrix (WHIM), the CRS‐R, and the more detailed Sensory Modality Assessment and Rehabilitation Technique (SMART), which complement each other, were recommended by the UK guidelines (Royal College of Physicians 2022). The advanced brain imaging and electrophysiology techniques have provided a tremendous boost to DoCs patients. However, the techniques were required to be interpreted whether they have any prognostic value.

### 4.2 Establishing a Multidisciplinary Team for Individualized Training Support

Regarding the second aspect, a multidisciplinary rehabilitation team should be formed. This level of evidence is high as it was widely used in clinical work and showed a positive effect on patients. Multidisciplinary resource integration and cooperation between different disciplines are an important part of shortening the patient's recovery time and optimizing consciousness rehabilitation. In clinical practice, clarifying the composition of the multidisciplinary team and division of responsibilities and emphasizing the importance of regular and timely communication for teamwork to maximize the team's role are important.

### 4.3 Scientific Implementation of Consciousness Intentions

Early effective stimulation was pivotal to improving consciousness in DoCs patients (Xiong et al. 2023). The intervention elevating the level of consciousness is the key to managing DoCs in patients with BI. Several studies indicated restorative (e.g., surgical intervention, physical factor therapy, hyperbaric oxygen therapy, and electromagnetic stimulation) and compensatory (e.g., nutritional goal management) interventions as powerful conscious interventions that can help patients improve their level of consciousness (Wang et al. 2020; Jilka et al. 2014; Kurada et al. 2019). However, the findings were from a low‐quality cohort study or expert consensus rather than meta‐analyses and large, randomized trials; hence, the quality of evidence is low. Thus, more researches are required in the future. Meanwhile, clinical care workers should be encouraged to receive professional training on consciousness rehabilitation to provide more specialized and comprehensive practice guidance to patients.

### 4.4 Paying Attention to Sensory Stimulation Programs

Brain excitability improved after the stimulation of deep and shallow sensations, especially proprioception (Giacino et al. 2020). As strong scientific evidence, from a high‐quality meta‐analysis, sensory stimulation showed the benefit of improving consciousness in DoCs patients (Zuo et al. 2021). Stimuli including the patient's name or narratives in a familiar voice or music triggered stronger responses than neutral stimuli (Jain and Ramakrishnan 2020). In addition, tactile stimulation combined with auditory or multisensory stimulation was associated with better outcomes than using only auditory stimulation (Jain and Ramakrishnan 2020). The existing heterogeneities, such as different clinical settings, disease stages, and levels of consciousness impairment, as well as the modality, contents, frequency, and intensity of stimulation, eventually lead to an incompatibility effect (Li et al. 2020; Zhong et al. 2021).

### 4.5 Drug Program

Drug application was also an important aspect of improving the level of consciousness. Traditional therapeutic strategies prioritize the protection of the injured brain cranial nerves to decrease secondary injury, lesion size, and neuronal death (Hiskens 2022). Evidence supports that amantadine (200–400 mg/day) improves cognition and arousal after traumatic BI, recommended it in patients with severe TBI (China Association of Rehabilitation of Disabled Persons, China Association of Rehabilitation Medicine, China Rehabilitation Research Center 2023; Ni et al. 2018). Other drugs acting on dopaminergic, catecholaminergic, and cholinergic systems, as well as zolpidem and baclofen, were also reported to have clinical applications, but more evidence‐based medical evidence is needed (Giacino, Katz, and Schiff 2019). Ghiam et al. (2021) reported that *N*‐acetyl cysteine (NAC), minocycline (MINO), and (−)‐phenserine (PHEN) may serve as useful repositioned therapeutics in treating TBI, in which MINO was effective in combination with NAC in preventing oligodendrocyte damage. Maintaining an adequate level of sedation and analgesia plays a key role in the management of TBI. Therefore, when using tranquilizers to treat agitation, starting at low levels, proceeding slowly, and monitoring effects on agitation and consciousness are recommended to avoid negative effects on brain function (Godoy et al. 2021). Despite numerous preclinical studies that identified promising pharmacologic treatments, all Phase III clinical trials currently provide unclear findings regarding which drug or combination of drugs is most effective in improving the level of consciousness, while no drug has been approved by the Food and Drug Administration (FDA) for TBI treatment (Hiskens 2022; Ismail et al. 2020).

### 4.6 Rehabilitation Training

The disuse syndrome occurs commonly in pDoCs patients, manifesting as cognitive impairment, muscle weakness, and ankyloses. Chinese Neurosurgical Society, China Neurosurgical Critical Care Specialist Council (2020) reported that early rehabilitation and nursing care can maximize cognitive and functional recovery, reduce complications, shorten hospitalization duration, reduce the financial burden on patients, and help them return to their families and society as soon as possible. Passive rehabilitation training or neuromuscular electrical stimulation should be performed as early as possible after stabilizing the condition. Furthermore, active training should be assisted as early as possible for patients with partial recovery of consciousness (Tramontano et al. 2022). However, Formisano et al. (2017) pointed that the better recovery and more positive outcome as resulting from early rehabilitation may be due to less severity of BI and fewer complications in the acute and postacute phase than to when the rehabilitation starts. The current evidence supports multitasking training rather than a program limited to a single task. It also supports early and long training, with some therapist's support. However, providing strong recommendations regarding the preference for a specific rehabilitation program is currently not possible. The influence of rehabilitation parameters (training duration and therapist's involvement) remains difficult to assess (Mazo et al. 2024).

### 4.7 The Prevention and Management of Complications

Close monitoring and expert medical care were urgently needed in DoCs patients due to the high rate of medical complications (Giacino et al. 2020). The prevention and management of complications are the key to long‐term survival for pDoCs patients. The rate of recovery from VS diminishes significantly over time. The odds of regaining consciousness were at least 54% upon admission but decreased to 48%, 33%, 19%, and 7% at 3, 6, 9, and 12 months after admission, respectively (Aidinoff et al. 2020).

For patients with a poor prognosis, long‐term nursing must be emphasized (Giacino et al. 2018; Raciti et al. 2021). A substantial amount of literatures indicated that routine nursing care includes position management (regular turning to avoid pressure sores), airway care (keeping the airway open; maintaining airway humidification; turning and knocking on the back; and assisting in sputum expulsion by using postural drainage, assisted sputum expectoration, and prone ventilation), nutritional screening (assisting in the selection and maintenance of nutritional pathways), oral care (maintaining oral hygiene), and deep vein thrombosis assessment (Group of Disorders of Consciousness and Conscious‐Promotion, Professional Committee of Neurorepair of Chinese Medical Doctor Association 2020; Raciti et al. 2021; Pan et al. 2021). However, standards of care have not yet been established to guide rehabilitative treatment in patients with DoCs. Without clear‐cut guidelines, considering and developing these interventions as an integral part of the multidisciplinary management of patients with DoCs should be interesting and helpful.

### 4.8 Limitations

First, only Chinese and English articles were included in this study, excluding literature written in other languages. Second, the effect of evidence application may have deviated from the actual situation in diverse cultural, regional, and racial backgrounds. Third, our study focuses more on the state of consciousness (i.e., the ability to awaken or conscious state) while neglecting the content of consciousness (i.e., cognitive function, memory, sensory integration, and higher brain functions) according to classical neuroscience theories.

## 5 Conclusion

This study summarizes the evidence for improving and managing of awareness in patients with critical BI, providing a reference for relevant clinical practice. The 26 pieces of evidence were chosen. However, as those studies were from different countries, relevant factors such as the clinical equipment, environment, and disease severity should be evaluated before implementing the evidence into practice (Farisco et al. 2024). Currently, high‐quality RCTs are rare in this area; hence, further studies should be monitored to extend the available knowledge. Meanwhile, future studies should focus on more specific family‐centered sensory and affective stimulation such as virtual reality.

## Author Contributions

**Miaoyuan Lin**: writing–review and editing, formal analysis, software, data curation, conceptualization, investigation. **Qiongna Lu**: investigation, writing–review and editing. **Sheng Yu**: investigation, visualization. **Wenjuan Lin**: conceptualization, supervision. All authors have critically discussed the results and approved the final manuscript.

## Conflicts of Interest

The authors declare no conflicts of interest.

### Peer Review

The peer review history for this article is available at https://publons.com/publon/10.1002/brb3.70260.

## Data Availability

Data sources UpToDate, BMJ Best Practice, Guideline International Network, the Cochrane Library, Embase, PubMed, Sinomed, Web of Science, CNKI, WanFang Database, and Chinese Medical Journal Full‐Text Database were searched from January 2018 to January 2024.
